# Cancer risk factors in systemic lupus erythematosus: multivariate regression analysis in 16,409 patients

**DOI:** 10.1186/ar4651

**Published:** 2014-09-18

**Authors:** Sasha Bernatsky, Rosalind Ramsey-Goldman, Jean-François Boivin, Lawrence Joseph, Michelle A Petri, Asad Zoma, Susan Manzi, Murray B Urowitz, Dafna D Gladman, Paul R Fortin, Ellen M Ginzler, Edward Yelin, Sang-Cheol Bae, Daniel J Wallace, Steven M Edworthy, Soren Jacobsen, Caroline Gordon, Mary A Dooley, Christine A Peschken, John G Hanly, Graciela S Alarcón, Ola Nived, Guillermo Ruiz-Irastorza, David Isenberg, Anisur Rahman, Torsten Witte, Cynthia Aranow, Diane L Kamen, Kristján Steinsson, Anca Askanase, Susan G Barr, Lindsey A Criswell, Gunnar Sturfelt, Neha M Patel, Jean-Luc Senécal, Michel Zummer, Janet E Pope, Stephanie Ensworth, Hani El-Gabalawy, Timothy McCarthy, Yvan St Pierre, Anne E Clarke

**Affiliations:** 1 McGill University Health Centre, Montreal, QC, Canada; 2 Northwestern University Feinberg School of Medicine, Chicago, IL, USA; 3 McGill University, Montreal, QC, Canada; 4 Johns Hopkins University School of Medicine, Baltimore, MD, USA; 5 Hairmyres Hospital, East Kilbride, UK; 6 West Penn Allegheny Health System, Temple University, Pittsburgh, PA, USA; 7 Toronto Western Hospital, Toronto, ON, Canada; 8 Université Laval, Canada; 9 State University of New York, Downstate Medical Center, Brooklyn, NY, USA; 10 University of California, San Francisco, CA, USA; 11 Hanyang University Hospital for Rheumatic Diseases, Seoul, Korea; 12 Cedars-Sinai Medical Center/David Geffen School of Medicine at UCLA, Los Angeles, CA, USA; 13 University of Calgary, AB, Canada; 14 Rigshospitalet, Copenhagen University Hospital, Copenhagen, Denmark; 15 University of Birmingham, College of Medical and Dental Sciences, Birmingham, UK; 16 University of North Carolina at Chapel Hill, NC, USA; 17 University of Manitoba, Winnipeg, MB, Canada; 18 Dalhousie University and Capital Health, Halifax, NS, Canada; 19 The University of Alabama, Birmingham, AL, USA; 20 Lund University Hospital, Lund, Sweden; 21 Rheumatic Diseases Research Unit, Bizkaia, Spain; 22 University College, London, UK; 23 Hannover Medical School, Hannover, Germany; 24 The Feinstein Institute for Medical Research, Manhasset, NY, USA; 25 Medical University of South Carolina, Charleston, SC, USA; 26 Landspitali University Hospital, Reykjavik, Iceland; 27 New York University School of Medicine, New York, NY, USA; 28 Université de Montréal, QC, Canada; 29 University of Western Ontario, London, ON, Canada; 30 University of British Columbia, Vancouver, BC, Canada

## Background

Patients with systemic lupus erythematosus (SLE) experience an increased risk of cancer, which is particularly driven by hematological malignancies. Our objective was to determine whether certain factors (demographics, SLE duration and calendar year) were associated with cancer risk in SLE, relative to the general population, using a large multicenter clinical cohort.

## Methods

We present detailed analyses of a multisite international SLE cohort (30 centers, 16,409 patients). Cancers were ascertained by registry linkage. Standardized incidence ratios (SIR; ratio of observed to expected cancers) were calculated for overall and for hematological cancer risk, representing the relative risk of cancer for SLE patients, versus the age, sex, and calendar-year-matched general. We used Poisson hierarchical regression to assess for potential independent effects of the factors examined (sex, race/ethnicity, age group, SLE duration, calendar-year period) on the SIRs among the SLE cohort members. The hierarchical nature of the model allowed for differences in effects from one country to the next.

## Results

In adjusted analyses (Table 1), we demonstrated lower SIR estimates for overall cancer risk, in black versus white SLE patients, in SLE patients of older versus younger age, and for patients with SLE duration of 5 years or more (versus lower duration). Female sex and calendar year were not clearly associated with any differences in the SIR estimates for SLE patients. Regarding hematological cancer specifically, SLE duration of 5 years or more again appeared to be associated with lower SIR estimates.

**Table 1:** Results of adjusted multivariate regression to determine independent effect of variables on SIR* estimates for cancer in SLE

	**Adjusted effects**^a^	95% confidence interval
Female sex	1.00	0.77 to 1.30
Race/ethnicity		
White	Reference group	
Black	0.75	0.58 to 0.97
Asian	1.18	0.85 to 1.62
Age		
<40	Reference group	
40 to 59	0.72	0.55 to 0.94
60+	0.55	0.42 to 0.73
SLE duration		
<5 years	Reference group	
≥5 years	0.74	0.61 to 0.89
Calendar-year period		
<2000	Reference group	
≥2000	0.95	0.79 to 1.15

^a^Variables adjusted concomitantly for all others (sex, race/ethnicity, age, SLE duration, calendar-year period).

## Conclusions

Although cancer risk in SLE is increased relative to the general population, patients most at risk appear to be those of white race/ethnicity, younger age, and of shorter SLE duration.

